# Enhancement of Starch Gel Properties Using Ionic Synergistic Multiple Crosslinking Extrusion Modification

**DOI:** 10.3390/foods13010024

**Published:** 2023-12-20

**Authors:** Wenguang Wei, Min Wu, Tianqi Zhang, Xun Zhang, Weike Ren, Tao He

**Affiliations:** College of Engineering, China Agricultural University, No. 17 Qinghua East Road, Haidian District, Beijing 100083, China

**Keywords:** cationic synergistic, multiple crosslinking, starch modification, rheological properties, extrusion

## Abstract

Crosslinking is a promising method to modulate the gel properties of food-grade starch gels. Still, the poor crosslinking effect of a single type of crosslinker limits the application of this method in starch gel modification. In this study, an Ca^2+^ synergistic multiple crosslinking modification method was proposed to prepare crosslinked starches with good gel properties and setting. The rheological properties of the samples were tested. The modified sample (SC-Ca-N3, G′ = 1347 ± 27) showed a 79% improvement compared to the starch without synergistic crosslinking modification (SC-N, G′ = 752 ± 6). The elastic modulus of starch gels can be adjusted by changing the degree of the crosslinking reaction. The results of nonlinear rheological Lissajous curve analysis showed that the synergistically crosslinked gel system strongly resisted deformation. In addition, the microstructure of the modified samples was characterized using scanning electron microscopy. The XPS, FTIR, and XRD results indicated that multiple molecular forces participate in the synergistic crosslinking reaction.

## 1. Introduction

Starch-based gels have a wide range of applications in the food, biological, and pharmaceutical fields due to their advantages in terms of low cost, biodegradability, safety, and edibility, and they are receiving increasing attention. However, natural starch gels are unstable and poorly adjustable in their rheological properties and viscosity due to the wide variation in the quality of natural starch raw materials and poor controllability, resulting in the preparation of natural starch gels that are not able to meet the differentiated demands for gel material properties in various fields [1,2,3]. Chemical modification is a compelling technical way to enhance the stability and setting of the rheological properties of natural starch gels. Among them, crosslinking modification has been emphasized by researchers due to its advantages of having a low cost and simple reaction process [3,4].

Crosslinking refers to the reaction in which crosslinker molecules connect adjacent starch molecules through crosslinking bonds generated with esterification and etherification reactions with free hydroxyl groups on the long chains of different starches, thus enhancing the overall properties of starch gels. Crosslinkers can be classified into esterification crosslinkers and etherification crosslinkers according to the type of crosslinking bonds generated [5].

To further improve the degree of crosslinking modification, the researchers tried to combine different types of crosslinkers to perform crosslinking modification. However, unfortunately, the crosslinking effect was not satisfactory [5,6,7]. The poor crosslinking may be due to the dense shell structure of starch granules limiting the rapid entry of crosslinking molecules, which inhibits the crosslinking efficiency; in addition, the competitive relationship between the molecules of esterified and etherified crosslinking agents inhibit each other, which also reduces the degree of the crosslinking reaction [8,9].

The study shows that the high-shear, high-pressure, and high-temperature environment in the barrel of the extruder can break the hard outer casing of starch granules, enhance the starch pasting efficiency and pasting degree, and realize the complete unfolding of starch long chains [10,11]. The fully unfolded starch long chains are favorable to react with free crosslinking molecules. In addition, some research has shown that ionic electrostatic interactions can effectively narrow the distance between the reacting molecules and promote the reaction [12,13]. Therefore, the hypothesis was proposed that the extrusion-ion synergistic modification method could effectively promote the multiple crosslinking reactions in the starch gel system and then realize the preparation of functional starch gels with good gel properties and regulatory properties.

In this study, the preparation of multi-crosslinked starch gels based on extrusion and ionic synergism was carried out by selecting citric acid as the esterifying crosslinking agent and dumbbell-type neopentyl glycol diglyceryl ether crosslinking agent (NGDE) and poly (ethylene glycol) diglyceryl ether (EGDE) as the etherifying crosslinking agent. The rheological properties were tested for dynamic detection and quantitative crosslinking reaction characterization. The modified gels were further characterized using SEM, XRD, infrared, etc., which revealed the microstructural and molecular reaction mechanisms based on the enhancement of the properties of modified samples.

## 2. Material and Methods

### 2.1. Material

Corn starch was purchased by LanYiYi Co. Ltd. (Beijing, China). CA, neopentyl glycol diglyceryl ether (NGDE), calcium citrate and poly (ethylene glycol) diglyceryl ether (EGDE) were provided from Sigma-Aldrich (St. Louis, MO, USA).

### 2.2. Preparation of Modified Samples

The preparation of a variety of multiple crosslinking solutions, the detailed composition ratio of the solution is shown in Table 1. Samples were prepared using a twin-screw extruder (TwinLab-F 20/40, Brabender, Oberhausen, Germany). Extruder barrel setting parameters were set: water feed 43.97 mL/min, feed 11.03 g/min, barrel temperature gradient (60, 80, 90, 90, 80 °C). The extrudates were control starch group (S) samples. The starch raw material was modified by extrusion using calcium citrate solution as a crosslinking agent to produce modified starch gel samples (S-Ca).

**Figure 1:**
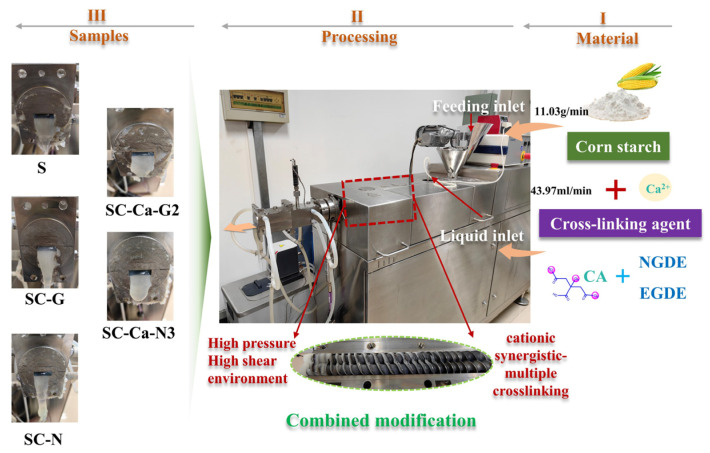
The preparation process of modified starch.

Similarly, without changing any other extruder settings, starch was sequentially modified with different types of crosslinker solutions to obtain the corresponding modified starch gel samples (SC-G, SC-N, SC-Ca-G1, SC-Ca-G2, SC-Ca-G3, SC-Ca-G4, SC-Ca-N1, SC-Ca-N2, SC-Ca-N3, SC-Ca-N4), respectively (Figure 1).

### 2.3. Rheological Characterisation

The rheological properties of the modified samples were tested using a rheometer TA ARES G2 (TA Instruments, Newcastle, DE, USA). The gap between the plates and the sample stage was 1 mm. The diameter of the parallel steel plates is 50 mm. Before starting the test, the gel was allowed to stand on the parallel plates at room temperature for 10 min to ensure the sample temperature was uniform in all areas. Strain-scanning tests determined the samples’ linear viscoelastic region (LVR) [14].

#### 2.3.1. Small-Amplitude Oscillatory Shear (SAOS) Test

The samples were subjected to a frequency scan (frequency 1–100 Hz, strain 0.5%). A power-law equation described the experimental results [15].

#### 2.3.2. Large-Amplitude Oscillatory Shear (LAOS) Test

Strain scans were performed on the samples (frequency 1 Hz, strain 0.1–1000%). Establishing the critical strain (γc) as the strain level at which G′ decreases by 5% within the LVR, and we concurrently recorded the corresponding loss modulus G″. We calculated the cohesive energy density (Ec). Lissajous curves were plotted using the MIT program (MIT beta). Lissajous curves help to visualize nonlinear behavior [14].

### 2.4. Determination of Degree of Crosslinking

A fast viscosity analyzer Fast-15 (Shanghai Baosheng Industrial Development Co., Ltd., Shanghai, China) was used to calculate the relative crosslinking, and the relative degree of crosslinking (Lc) was determined [7].

### 2.5. Fourier Transform–Infrared (FT-IR-ATR) Spectroscopy

FTIR analysis was performed in triplicate for samples using an FT-IR spectrophotometer-equipped Spectrum 100 (Thermo Fisher Scientific, Norwalk, CA, USA) with attenuated total reflection (ATR) [16].

### 2.6. Diffraction of X-rays (XRD)

The samples were analyzed using X-ray diffraction BJX-2D (BGOL, Beijing, China) (2θ: 5°~50°). MDI Jade5.0_v5.0.37 software was used to calculate the relative crystallinity (RC) The Savitzky–Golay algorithm was used to further smooth the diffraction curve. Relative crystallinity values were calculated using the ratio of crystalline area to total peak area as represented [17].

### 2.7. Scanning Electron Microscopy (SEM)

The samples were photographed using scanning electron microscopy. In brief, starch gel samples were soaked in alcohol, and then they were quickly frozen at −80 °C for a whole day. After that, the divided frozen gels were vacuum dried. The appropriate size was sprayed with gold using a 10 KV ion sputter coater SBC-12 (KYKY Co., Ltd., Beijing, China). The samples were placed in SEM SU3500 (Hitachi High-Tech Co., Ltd., Tokyo, Japan) for observation. To quantitatively analyze the differences in SEM micrographs, the microstructure characteristics were measured using Image-J v1.52i [18].

### 2.8. Thermal Properties

A DSC analyzer Model 60A (Shimadzu Corporation, Kyoto, Japan) was used to analyze the thermal properties of the samples. A total of 2 mg (dry weight) of the sample was accurately weighed into an aluminum pan and mixed with deionized water (1:10, *w*/*w*). The pan was sealed and then heated from 20 °C to 120 °C at a rate of 5 °C/min. An aluminum pan was used as a reference. [19]. Thermogravimetric analysis (TGA) of crosslinked modified samples tested using the method of Wu et al. [20] was performed using a TGA thermogravimetric analyzer TGA5500 (TA Instruments, Newcastle, DE, USA). The weight of the samples was 10 mg, and the heating-temperature interval was from 30 °C to 800 °C. The heating process was protected by a constant flow of nitrogen gas [20].

## 3. Results and Discussion

### 3.1. Rheological Characterisation

#### 3.1.1. Flow Behavior

**Figure 2:**
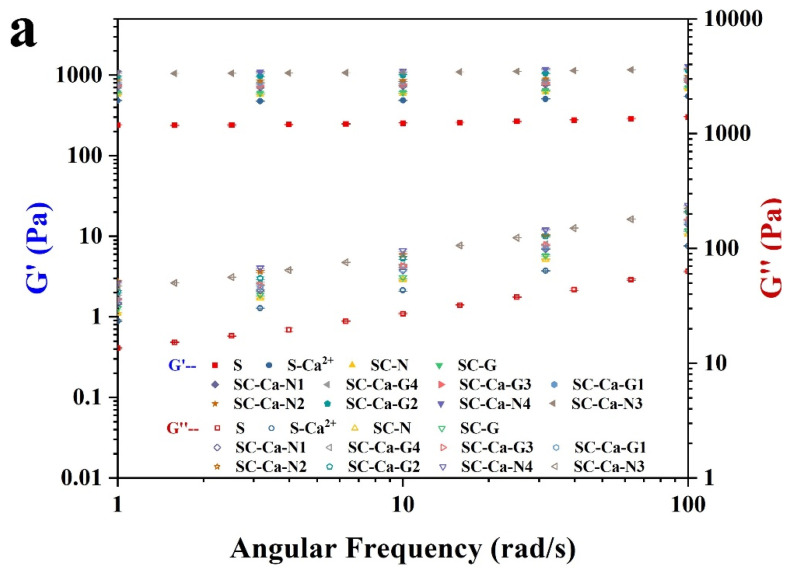

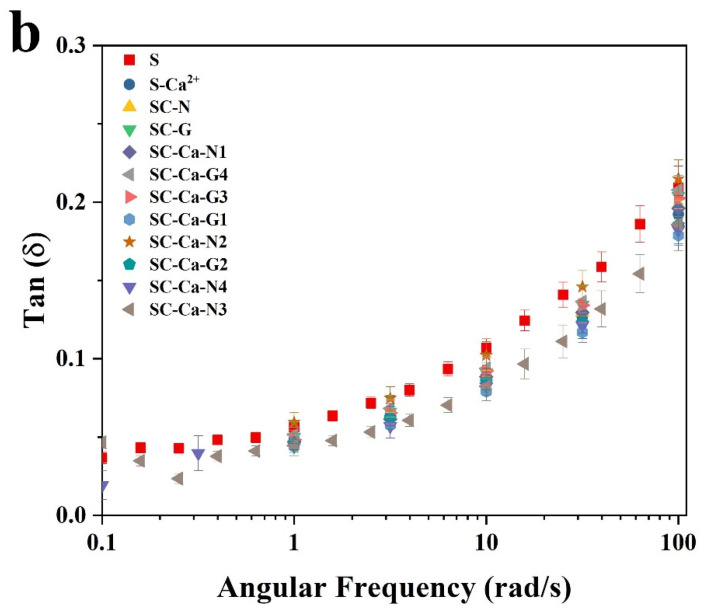
The storage modulus G′, loss modulus G″ (**a**), and loss factor Tanδ (**b**).

In order to investigate the effect of modification on the texture and structural characteristics of the starch hydrogels, the samples were subjected to frequency scanning (Figure 2a,b) [14]. As shown in Figure 2a, the unmodified sample S exhibited the lowest storage modulus (G′), and the samples (SC-N and SC-G) with the addition of the esterified–etherified dual crosslinker system showed a slight enhancement in G′. This may be because the esterified and etherified crosslinker molecules compete for crosslinking sites and thus inhibit the overall crosslinking reaction of the sample. Notably, there is a significant enhancement in the G′ of the starch gel samples after adding charged metal ions to the esterified–etherified dual crosslinker system. The ion synergistically double crosslinking modified SC-Ca-N3 and SC-Ca-G2 showed the highest G′ in their series of samples, 452 ± 7% and 405 ± 5% of the G′ of the unmodified sample S, respectively. It can be shown that the metal ions have a synergistic enhancement effect on the esterification–etherification double crosslinking reaction, and the enhancement may be related to the electrostatic interaction force between the Ca^2+^ and the crosslinker molecules [21,22]. In addition, the G′ of SC-Ca-N4 is reduced compared to SC-Ca-N3; similarly, the G′ of SC-Ca-G3 and SC-Ca-G4 is reduced compared to SC-Ca-G2. This suggests that the excessive addition of etherified crosslinkers can disrupt the ionic synergistic reinforcement effect, reducing the overall degree of crosslinking.

Furthermore, the storage modulus (G′) was greater than the loss modulus (G′) for all gel samples, demonstrating that the starch gels had a solid gel structure. The tanδ values were used to comprehensively assess the viscous and elastic behavior of the samples (Figure 2b). The results showed that the tanδ values ranged between 0.02 and 0.25, further confirming that the starch had transformed into a solid gel state [23]. The frequency dependence of the samples’ G′ and G″ before and after modification was quantified through fitting a power law model (Table 2) and was expressed in terms of n′ and n″, respectively. The SC-Ca-N3 had the lowest n′ value, indicating that the ionic synergistic double crosslinking reaction reduced the starch gel’s frequency dependence. This result also confirms that the ionic synergistic modification produced a stronger crosslinked structure in the gel and suppressed an effect of shear on the gel structure in LVR [15].

#### 3.1.2. Strain Amplitude

**Figure 3:**
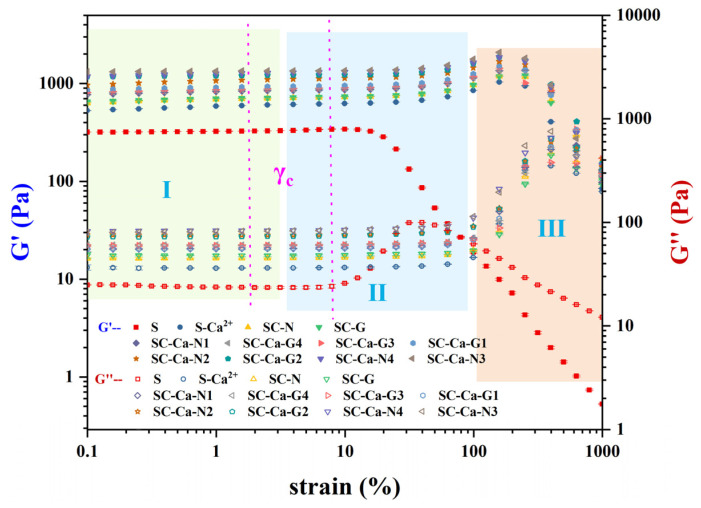
The high-amplitude oscillatory shear behavior of starch gel samples.

The G′ and G″ of the gel samples are preserved as stable and unchanged in LVR (Figure 3, I). G′ > G″ indicates that the elastic behavior of the sample dominates. The modified samples showed significantly higher γc and extended LVR than the control sample S (γc = 0.7 ± 0.3) (Table 3). In addition, the most significant increase in γc was observed for SC-Ca-N3 (γc = 3.2 ± 0.3), confirming that the gel system modified with ionic synergistic double crosslinking has a strengthened network structure and exhibits better strain resistance [14,24]. When the strain exceeds γc, the sample gel exhibits a common type III rheological behavior [23]. This is because the network structure that maintains its overall elasticity in the sample gel under medium amplitude oscillatory shear conditions breaks down rapidly. At this point, the shear energy is converted further into loss modulus, and there is an upsurge in the disassembly and rearrangement of molecular clusters in the sample, resulting in G′overshoot behaviour [13,15,25]. As the strain amplitude increases further, the gel network structure is completely torn, leading to “liquefaction” of the hydrogel (Figure 3, III) [15].

The ionic synergistic double crosslinking reaction affects both the linear and nonlinear viscoelastic rheological properties of starch gel systems, which may be related to forming a tighter and smaller network structure in the synergistically crosslinked gels [22,26].

### 3.2. Determination of Degree of Crosslinking

Samples with the double crosslinking agent added (SC-G, SC-N) had a lower degree of crosslinking of 5.3 ± 0.2 and 5.6 ± 0.3, respectively (Table 3). However, the *Lc* of SC-Ca-G3 and SC-Ca-N3 was significantly increased to 13.5 ± 0.1 and 19.2 ± 0.1. The above results confirm that adding Ca^2+^ can improve the crosslinking of the starch [7,15]. In addition, the relative degree of crosslinking of the samples is consistent with the changes in G′ and G″ of the samples, which confirms that the changes in the rheological properties of the samples in Section 3.1 are related to the degree of crosslinking.

### 3.3. Fourier Transform–Infrared (FT-IR) Spectroscopy

**Figure 4:**
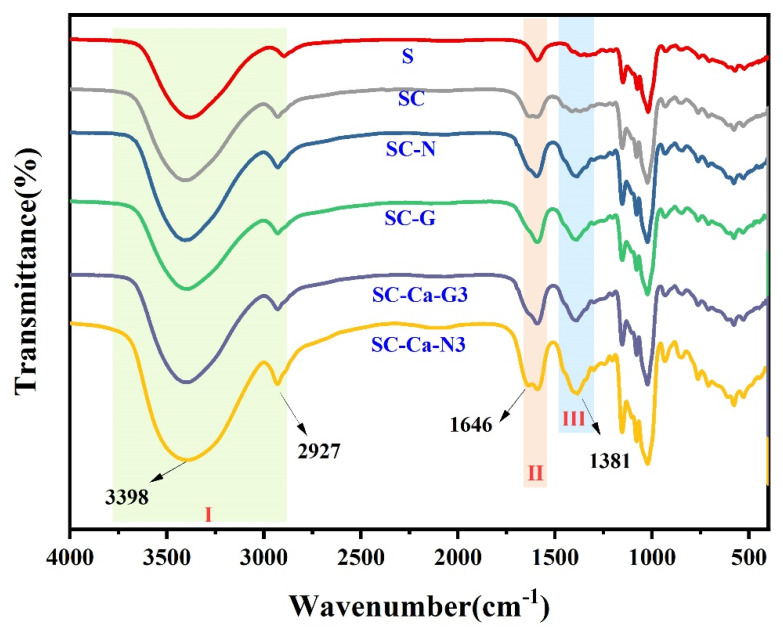
FITR-ATR of starch gels.

FTIR-ATR was used to determine the changes in the functional groups of the crosslinked modified starch gels. As shown in Figure 4, The broad peak at 3300–3700 cm^−1^ is assigned to the stretching vibration of hydroxyl (–OH) groups. The absorption peak at 2926 cm^−1^ is due to the C–H stretching (Figure 4, I). The peak at 1616 cm^−1^ is due to the bound water. The SC, with the addition of citric acid, showed a new peak at 1646 cm^−1^ compared to unmodified starch S. This peak confirms the formation of carboxyl and ester carbonyl bond formation [16]. This indicates that citric acid and starch undergo an esterification reaction to form crosslinking bonds. With the addition of an etherified crosslinking agent, SC-G and SC-N showed a new peak at 1381 cm^−1^ (Figure 4, III), which was attributed to the formation of an ether bond [27]. At the same time, the intensity of the characteristic peaks of the sample’s ester group was weakened (Figure 4, II), confirming the existence of antagonistic effects between different types of crosslinking agents during the crosslinking reaction. This antagonistic effect reduces the overall degree of the crosslinking reaction of the sample. This conclusion is consistent with the pattern of change in the gel properties of the samples in Section 3.1. Notably, after the introduction of cations, the characteristic peaks of the ester group and ether bond of both SC-Ca-N3 and SC-Ca-G3 were significantly enhanced. This suggests that cationic synergistic can effectively promote the esterification–etherification double cross-coupling reaction [28,29].

### 3.4. X-ray Diffraction (XRD) Analysis

**Figure 5:**
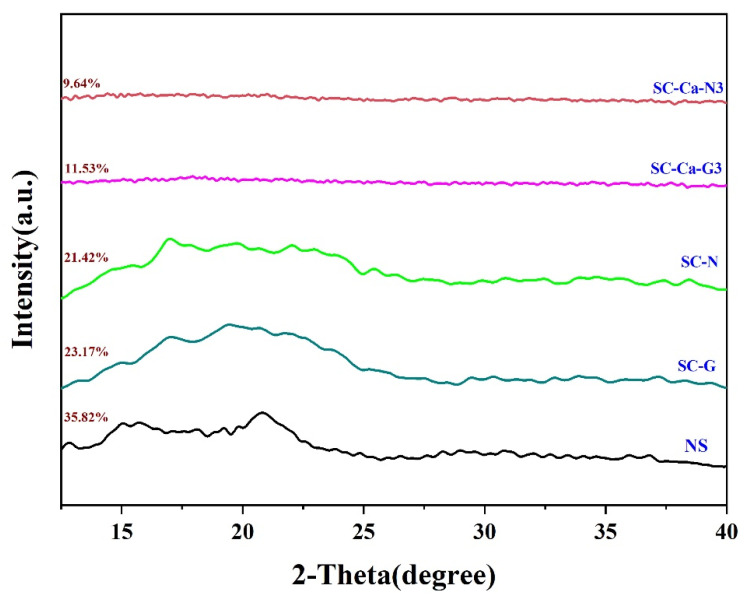
XRD of starch gels.

As shown in Figure 5, native regular corn starch displayed a distinctive A-type XRD pattern with strong diffraction peaks at around 15° and 23° 2θ and an unresolved doublet at around 17° and 18° 2θ [30]. In contrast, the crystal structure of the starch gel sample S, which had high-temperature pasting during the extrusion process, was destroyed, and its initial diffraction peak at 2θ = 12.7° disappeared [31]. The SC-N and SC-G with the addition of double crosslinkers showed broad peaks at 2θ = 14°–26° and lost the crystalline peaks usually associated with natural starch, suggesting that their structures are characterized as amorphous [17,27]. Based on the subsequent estimation of the crystallinity, the starch sample SC-Ca-N3 modified using ionic synergistic double crosslinking showed the lowest crystallinity. This phenomenon can be explained by the higher degree of crosslinking of the double crosslinking modification through ion synergism, which restricts the hydrogen-bonding interactions between the chains of starch molecules and hinders the orderly arrangement of these chains during the crystallization process, ultimately leading to a lower degree of crystallinity of SC-Ca-N3 [20,27,32].

### 3.5. Microstructure Analysis

**Figure 6:**
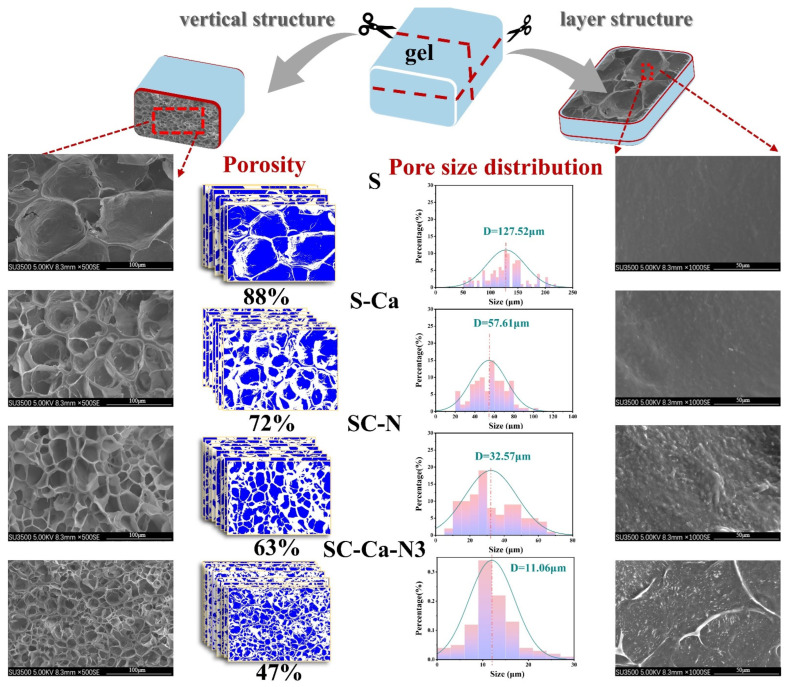
SEM images; porosity and pore size distribution.

Modifications effect the microstructural properties of samples [18]. Therefore, the samples’ longitudinal structural characteristics and transverse structural characteristics were investigated using scanning electron microscopy (SEM), as shown in Figure 6. The starch gels exhibited a porous network structure in the longitudinal direction. In addition, the pore sizes of the S-Ca, SC-N, and SC-Ca-N3 samples were larger than those of S. The pore size of SC-Ca-N3 was 11.06 μm, and its micropores were the densest. This observation may be the result of the ionic synergistic effect that significantly enhances the double crosslinking reaction, increasing the number of attachment sites between starch chain segments and leading to a dense microporous network structure [33]. Meanwhile, the porosity of SC-Ca-N3 was reduced (47 ± 2%). Decreased porosity means a denser porous structure and greater toughness [34]. The surface of the transverse structure exhibits different structural characteristics from the longitudinal direction. The surface structure of gel S is flat. With the addition of Ca^2+^, microporous protrusions appeared on the layer surface. However, SC-Ca-N3 has interconnected dense and porous fibrous network-like protrusions. This result may be due to the formation of a flat structure of the starch chains in S through the interaction of hydroxyl groups [35]. In contrast, SC-Ca-N3 contains many crosslinking bonds between the starch chains, which impede the interactions between the starch chains, hindering the movement and stacking of these chains and ultimately forming a porous mesh surface [36,37]. The ionic synergistic double crosslinking reaction promotes the formation of dense gel reticular porous structures. These dense porous structures provide a stronger force-supporting structure for the gels, which appropriately explains the microscopic reason for the excellent elastic modulus of SC-Ca-N3 in Section 3.1.

### 3.6. Thermal Performance Analysis

**Figure 7:**
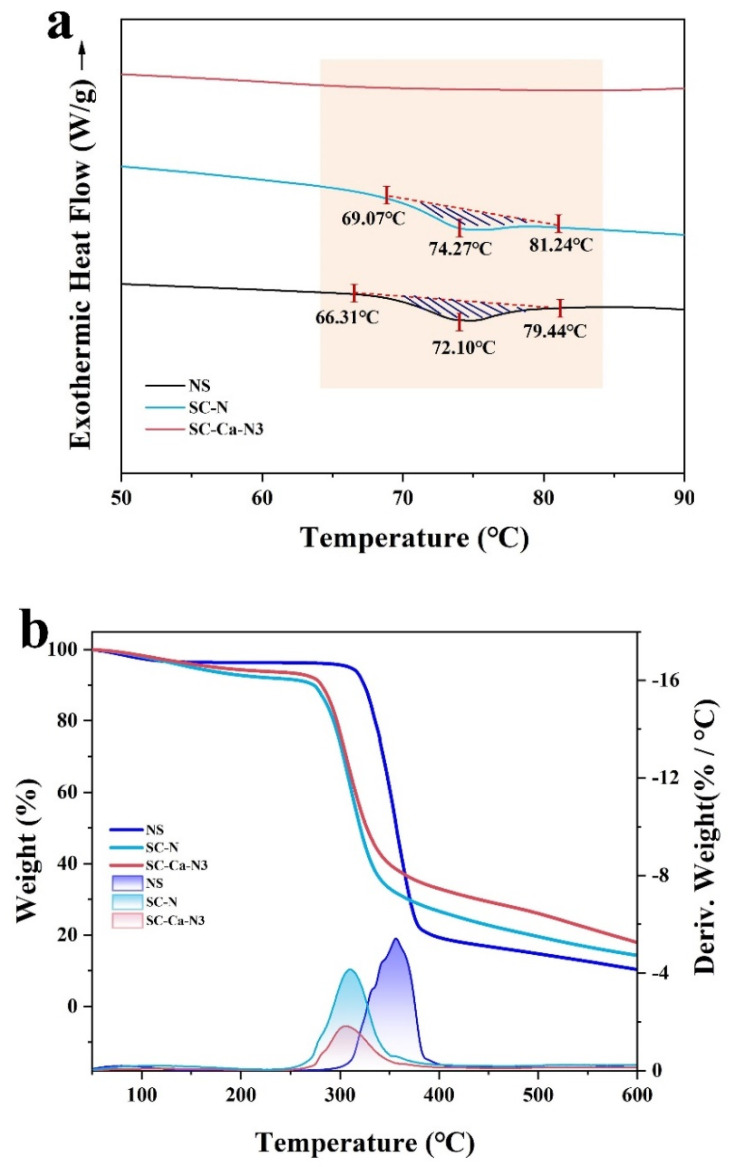
DSC (**a**) and TG (**b**) thermograms.

DSC and TG analyses investigated the characteristics of the multiply crosslinked starches. The NS exhibited a single-peak pasting profile at 72.10 ± 0.08 °C (Figure 7a) [38]. On the contrary, the pasting peak of SC-N shifted towards higher temperatures and appeared at 74.27 ± 0.06 °C. Research has shown that enhanced double helixes require higher temperatures for pasting [19,39]. The paste peak of the synergistically crosslinked SC-Ca-N3 disappears (Table 4). The total ΔH of SC-N was significantly reduced from 16.82 ± 0.4 (natural starch) to 13.14 ± 0.08 J/g (*p* < 0.05) compared to natural starch. The enthalpy of pasting indicates the energy required for the melting of double helices, and the decrease in the enthalpy of pasting suggests that double crosslinking dramatically reduces the crystal structure in starch [40,41,42]. Hydration and swelling of amorphous regions in starch granules promotes crystallization melting. Ionic synergistic dual crosslinking modification inhibits starch granule swelling, prevents melting of starch junction regions, and leads to the disappearance of the pasty peak [38,40,43].

The thermal decomposition process is divided into three main phases from 25 °C to 800 °C (Figure 7b). The first region in between 51 and 150 °C originates from the removal of water in the sample [44]. The second weight-loss peak is attributed to the decomposition of esterified and etherified crosslinkers in the sample, occurring from 180 °C to 235 °C [27,45]. The third region in between 270 °C and 385 °C originates from the pyrolysis of starch molecules [46]. The ionic synergistic double crosslinking reduced pyrolysis of SC-Ca-N3, indicates that the ionic synergistic double crosslinking modification enhanced the starch’s thermal stability. The results confirmed that the ionic synergistic crosslinking enhanced the intermolecular interactions of starch.

### 3.7. The Mechanism of Synergistic Modification

**Figure 8:**
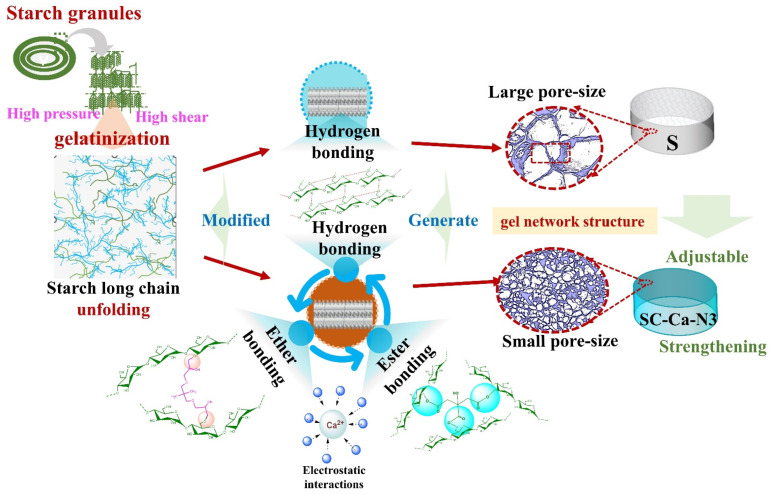
Mechanism of ionic synergistic multiple crosslinking on the regulation of starch gel properties.

The mechanism of ionic synergistic double crosslinking on the gels was further analyzed based on the experimental results, illustrated in Figure 8. The hydroxyl groups were exposed during the starch gelatinization process. The –COOH groups on the esterified crosslinker molecule crosslink with the surrounding free –OH groups through an esterification reaction and connect multiple long starch chains in its vicinity [47,48]. Meanwhile, the etherified crosslinker CA molecule contains epoxy groups that crosslink to the surrounding free hydroxyl groups via an etherification reaction [49]. Ca^2+^ cations that uniformly fill the gap between starch and free crosslinker molecules further connect unreacted free hydroxyl, carboxyl, and epoxy groups through electrostatic interactions. This process promotes the proximity of starch molecular chains and the reactive groups of the crosslinker [12,22,49]. As a result, the double crosslinking reactions in starch gels were promoted. As gelation occurs, starch molecular chains aggregate. The gel S collects and forms large pore-size cavities through hydrogen bonding [40,50].

The SC-Ca-N3 aggregate under the coupling of multiple intermolecular forces such as etherified crosslinking agents, esterified crosslinking bonds, etherified crosslinking agents, hydrogen bonding, and cationic electrostatic interactions, which results in the formation of a denser and more porous honeycomb network structure [51,52,53]. This dense and porous microstructural property affects the gel properties of modified starch. Meanwhile, flexible regulation of the gel properties can be realized by controlling the degree of crosslinking.

## 4. Conclusions

Crosslinked starch gels with enhanced and tunable gel properties were successfully realized using ionic synergistic multiple crosslinking extrusion modification. The effects of ionic synergistic multiple crosslinking on the gels’ rheological properties were investigated. The synergistic double crosslinking modification effectively improved the gel strength. The principle of the modification reaction was related to the formation of dense and porous microstructures in the gels. In addition, control of rheological properties could be realized by changing the degree of multiple crosslinking reactions. In addition, ionic synergistic multiple crosslinking improves the thermal stability, which is conducive to maintaining the textural properties.

## Figures and Tables

**Table 1:** The ratio of components in the crosslinking system of samples.

Sample	Molar Concentration Ratio of Each Component			
	Citric Acid	Calcium Citrate	Neopentyl Glycol Diglycidyl Ether (NGDE)	Polyethylene Glycol Diglycidyl Ether (PGDE)
S	0	0	0	0
S-Ca	0	0.5	0	0
SC-G	1	0	1	0
SC-N	1	0	0	1
SC-Ca-G1	1	0.5	0.6	0
SC-Ca-G2	1	0.5	0.8	0
SC-Ca-G3	1	0.5	1.0	0
SC-Ca-G4	1	0.5	1.2	0
SC-Ca-N1	1	0.5	0	0.6
SC-Ca-N2	1	0.5	0	0.8
SC-Ca-N3	1	0.5	0	1.0
SC-Ca-N4	1	0.5	0	1.2

**Table 2:** The fitting results of the frequency sweep.

Sample	G′= K′ω^n′^		*R* ^2^	*G*″ = K″ω^n″^		*R* ^2^
	K′	n′		K″	n″	
S	251 ± 5 ^a^	0.081 ± 0.006 ^a^	0.95	13.6 ± 0.5	0.63 ± 0.01 ^a^	0.92
S-Ca	484 ± 8 ^b^	0.073 ± 0.002 ^b^	0.95	19.3 ± 0.7	0.58 ± 0.01 ^b^	0.94
SC-G	577 ± 13 ^c^	0.068 ± 0.003 ^c^	0.96	23.1 ± 0.9	0.51 ± 0.03 ^c^	0.95
SC-N	612 ± 15 ^d^	0.063 ± 0.007 ^c^	0.93	24.5 ± 0.3	0.47 ± 0.02 ^c^	0.96
SC-Ca-N1	701 ± 16 ^e^	0.051 ± 0.006 ^d^	0.96	27.4 ± 0.1	0.40 ± 0.03 ^d^	0.92
SC-Ca-G4	739 ± 10 ^f^	0.048 ± 0.003 ^d^	0.91	31.3 ± 0.7	0.38 ± 0.02 ^d^	0.96
SC-Ca-G3	743 ± 14 ^g^	0.046 ± 0.005 ^d^	0.92	28.6 ± 0.6	0.35 ± 0.02 ^d^	0.91
SC-Ca-G1	796 ± 23 ^h^	0.042 ± 0.003 ^e^	0.92	30.2 ± 0.3	0.33 ± 0.01 ^e^	0.91
SC-Ca-N2	869 ± 27 ^i^	0.035 ± 0.002 ^f^	0.96	38.8 ± 0.5	0.28 ± 0.01 ^f^	0.96
SC-Ca-G2	1017 ± 34 ^j^	0.033 ± 0.002 ^g^	0.89	41.9 ± 0.9	0.25 ± 0.01 ^g^	0.89
SC-Ca-N4	1079 ± 13 ^k^	0.028 ± 0.002 ^h^	0.94	42.5 ± 0.4	0.21 ± 0.02 ^h^	0.86
SC-Ca-N3	1137 ± 21 ^l^	0.021 ± 0.001 ^i^	0.95	45.8 ± 0.1	0.12 ± 0.02 ^i^	0.96

*R*^2^ is the fit measure of the model; a–l lowercase letters indicate significant differences between samples (*p* < 0.05).

**Table 3:** Critical strain (γ_c_), G′ at critical strain (*G*_cr_), cohesive energy density (*E*_c_), and relative crosslinking degree (Lc).

Sample	Critical Strain *γ*_c_ (%)	G′ at Critical Strain *G*_cr_ (Pa)	Cohesive Energy Density *E_c_* (J/m^3^)	Lc (%)
S	0.9 ± 0.1 ^a^	324 ± 2 ^a^	0.01 ± 0.01 ^a^	0
S-Ca	1.5 ± 0.3 ^b^	495 ± 8 ^b^	0.06 ± 0.01 ^b^	1.1 ± 0.6 ^a^
SC-G	1.8 ± 0.2 ^b^	746 ± 6 ^c^	0.12 ± 0.01 ^c^	5.3 ± 0.2 ^b^
SC-N	2.0 ± 0.5 ^b^	752 ± 6 ^c^	0.15 ± 0.03 ^d^	5.6 ± 0.3 ^c^
SC-Ca-N1	3.2 ± 0.3 ^c^	893 ± 35 ^d^	0.46 ± 0.02 ^e^	9.3 ± 0.2 ^d^
SC-Ca-G4	3.5 ± 0.5 ^c^	924 ± 27 ^d^	0.57 ± 0.02 ^f^	11.6 ± 0.2 ^e^
SC-Ca-G3	3.7 ± 0.2 ^c^	931 ± 27 ^d^	0.64 ± 0.06 ^g^	13.5 ± 0.1 ^f^
SC-Ca-G1	4.1 ± 0.1 ^d^	953 ± 27 ^e^	0.81 ± 0.02 ^h^	14.2 ± 0.5 ^g^
SC-Ca-N2	4.4 ± 0.2 ^e^	1127 ± 27 ^f^	1.09 ± 0.05 ^i^	15.7 ± 0.3 ^h^
SC-Ca-G2	4.9 ± 0.3 ^f^	1247 ± 27 ^g^	1.49 ± 0.02 ^j^	17.1 ± 0.2 ^i^
SC-Ca-N4	5.7 ± 0.1 ^g^	1311 ± 27 ^h^	2.13 ± 0.03 ^k^	17.8 ± 0.2 ^j^
SC-Ca-N3	6.3 ± 0.3 ^h^	1347 ± 27 ^i^	2.67 ± 0.05 ^l^	19.2 ± 0.1 ^k^

a–l lowercase letters indicate significant differences between samples (*p* < 0.05).

**Table 4:** Thermal parameters of starch gels.

Samples	T_0_	T_p_	T_c_	T_c_–T_0_	ΔH
native starch	66.31 ± 0.08 ^a^	72.10 ± 0.03 ^a^	79.44 ± 0.06 ^a^	13.13 ± 0.02 ^a^	16.82 ± 0.04 ^a^
SC-N	69.07 ± 0.02 ^b^	74.27 ± 0.05 ^b^	81.24 ± 0.04 ^b^	12.17 ± 0.05 ^b^	13.14 ± 0.08 ^b^
SC-Ca-N3	ND.	ND.	ND.	ND.	ND.

a–b lowercase letters indicate significant differences between samples (*p* < 0.05). ND, not detected.

## Data Availability

Data are contained within the article.
